# Ketamine’s antidepressant effect is mediated by energy metabolism and antioxidant defense system

**DOI:** 10.1038/s41598-017-16183-x

**Published:** 2017-11-17

**Authors:** Katja Weckmann, Michael J. Deery, Julie A. Howard, Renata Feret, John M. Asara, Frederik Dethloff, Michaela D. Filiou, Jamie Iannace, Christiana Labermaier, Giuseppina Maccarrone, Christian Webhofer, Larysa Teplytska, Kathryn Lilley, Marianne B. Müller, Christoph W. Turck

**Affiliations:** 1 Max Planck Institute of Psychiatry, Department of Translational Research in Psychiatry, Munich, Germany; 2 Cambridge Centre for Proteomics, Cambridge System Biology Centre, University of Cambridge, Cambridge, UK; 3 Division of Signal Transduction, Beth Israel Deaconess Medical Center and Department of Medicine, Harvard Medical School, Boston, USA; 4 Max Planck Institute of Psychiatry, Department of Stress Neurobiology and Neurogenetics, Munich, Germany; 5 Experimental Psychiatry, Department of Psychiatry and Psychotherapy & Focus Program Translational Neuroscience, Johannes Gutenberg University Medical Center, Mainz, Germany; 6 Present Address: Institute of Pathobiochemistry, Johannes Gutenberg University, Medical School, Mainz, Germany

## Abstract

Fewer than 50% of all patients with major depressive disorder (MDD) treated with currently available antidepressants (ADs) show full remission. Moreover, about one third of the patients suffering from MDD does not respond to conventional ADs and develop treatment-resistant depression (TRD). Ketamine, a non-competitive, voltage-dependent N-Methyl-D-aspartate receptor (NMDAR) antagonist, has been shown to have a rapid antidepressant effect, especially in patients suffering from TRD. Hippocampi of ketamine-treated mice were analysed by metabolome and proteome profiling to delineate ketamine treatment-affected molecular pathways and biosignatures. Our data implicate mitochondrial energy metabolism and the antioxidant defense system as downstream effectors of the ketamine response. Specifically, ketamine tended to downregulate the adenosine triphosphate (ATP)/adenosine diphosphate (ADP) metabolite ratio which strongly correlated with forced swim test (FST) floating time. Furthermore, we found increased levels of enzymes that are part of the ‘oxidative phosphorylation’ (OXPHOS) pathway. Our study also suggests that ketamine causes less protein damage by rapidly decreasing reactive oxygen species (ROS) production and lend further support to the hypothesis that mitochondria have a critical role for mediating antidepressant action including the rapid ketamine response.

## Introduction

Mood disorders including major depressive disorder (MDD) are a leading cause of disability worldwide^1,2^. The prevalence for mood disorders has been estimated at 21 million people across 28 European countries with an annual cost of 106 billion Euro^3,4^. Globally, approximately 350 million people suffer from MDD, with women more likely to be affected than men^5,6^. The currently available antidepressants (ADs) require chronic long-term treatment of several weeks before a therapeutic effect is achieved^7,8^. Less than 50% of all patients treated with current ADs show full remission^9^. Moreover, about one third of the patients suffering from MDD do not respond to conventional ADs and suffer from treatment-resistant depression (TRD)^10^. Fifteen percent of severely depressed individuals commit suicide, the second leading cause of death for ages 15- to 29-years^6^.

In 2000 Berman *et al*. demonstrated for the first time a rapid antidepressant effect of a low dose of ketamine within 2 h and 4 h of treatment that lasts for up to 10 days^11^. Ketamine is a non-competitive, voltage-dependent N-Methyl-D-aspartate receptor (NMDAR) antagonist^12–17^. The antidepressant effect of ketamine is also observed in patients suffering from TRD^18,19^. Moreover, ketamine diminishes suicidal ideation^20^. However, psychomimetic side effects of ketamine have prevented its use as a first-line drug^21–25^.

A low dose of ketamine was initially reported to act through the activation of the mammalian target of rapamycin complex 1 (mTORC1) causing an increase of synaptic protein levels and an elevated number of spines and neuronal activity in the medial prefrontal cortex of rats^26^. Furthermore, increased levels of postsynaptic proteins required for formation, maturation, and function of new spines were observed 2 h after ketamine treatment and lasted up to 72 h^26^. More recent studies of ketamine’s antidepressant effects implicate a ketamine metabolite as the actual active compound. Results from studies in mice treated with the ketamine metabolite (2 R, 6 R)-hydroxynorketamine (HNK) suggest that the antidepressant-like effect of the drug is independent of NMDAR binding and implicate the α-amino-3-hydroxy-5-methyl-4-isoxazolepropionic acid receptor (AMPAR) and its signaling pathway to be critical for the activity^27^.

In previous studies we have shown an important role of mitochondrial energy metabolism for ketamine’s antidepressant mode of action. A time-dependent metabolomics profiling study implicated ‘glycolysis’ and the ‘citrate cycle’ pathways. In the present paper we report on an extension of our analyses on ketamine’s fast acting antidepressant activity that further support a critical role of mitochondrial pathways impacting cellular oxidative stress defense mechanisms^28^.

## Results

**Figure 1:**
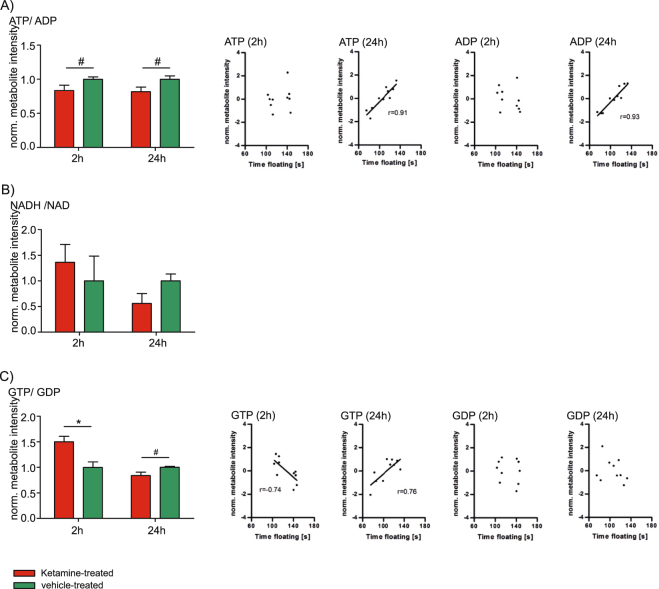
(**A**) Time-dependent hippocampal analysis of adenosine triphosphate (ATP)/adenosine diphosphate (ADP) metabolite ratio and correlation analyses of ATP (2 h, p ≥ 0.05 and 24 h, p ≤ 0.05) and ADP (2 h, p ≥ 0.05 and 24 h, p ≤ 0.05) with the forced swim test (FST) floating time. (**B**) Time-dependent hippocampal analysis of nicotinamide adenine dinucleotide (reduced) (NADH)/nicotinamide adenine dinucleotide (oxidized) (NAD) metabolite ratio. (**C**) Time-dependent hippocampal analysis of guanosine triphosphate (GTP)/guanosine diphosphate (GDP) ratio and correlation analyses of GTP (2 h and 24 h, p ≤ 0.05) and GDP (2 h and 24 h, p ≥ 0.05) after a single injection of a low dose of ketamine with FST floating time. The results from ketamine-treated animals are normalized to the ones obtained from vehicle treatment (mean value = 1). N = 5 mice per group and time point. ^#^p ≤ 0.10, *p ≤ 0.05. P-values were determined by Student’s t-test and significance analysis of microarrays and other omic-datasets (SAM). Error bars represent s.e.m. The correlation coefficient r was calculated by Pearson. The linear regression line is only shown for significant (p ≤ 0.05) correlation coefficients.

Our previous study on ketamine’s mode of action suggested an involvement of mitochondrial energy metabolism, a finding that is also supported by its link to glutamate neurotransmission. Glutamate is metabolized from α-ketoglutarate, a ‘citrate cycle’ metabolite, and can be recycled through this pathway. Interestingly, glutamate neurotransmission also stimulates glucose utilization and ‘glycolysis’, the upstream pathway of the OXPHOS pathway. Therefore, we investigated whether ketamine’s fast antidepressant-like effects are reflected by metabolite ratios that are part of the ‘citrate cycle’ and the connected OXPHOS pathway, including ATP/ADP, NADH/NAD and GTP/GDP ratios. In addition, we correlated these metabolites with antidepressant-like behavior using the FST floating time as a readout. Interestingly, the ATP/ADP metabolite ratio tended to be decreased 2 h and 24 h after a single injection of a low dose of ketamine. In addition, ATP and ADP levels significantly and strongly correlated with the FST floating time at 24 h (Fig. 1A). ATP is produced from ADP by OXPHOS complex V and NADH serves as an electron donor for OXPHOS complex I^28,29^. The NADH/NAD ratio neither showed a significant difference nor a significant correlation with the FST floating time (Fig. 1B). GTP is synthesized from GDP as a by-product of succinate-CoA to succinate conversion in the ‘citrate cycle’. Succinate is an electron donor for the OXPHOS complex II subunit A that we have previously shown to be elevated 2 h after ketamine treatment^28^. We found that the GTP/GDP metabolite ratio is indeed significantly elevated 2 h upon ketamine treatment and tended to be downregulated at the 24 h time point. In addition, GTP levels significantly correlated with FST floating time at both time points (Fig. 1C).

**Figure 2:**
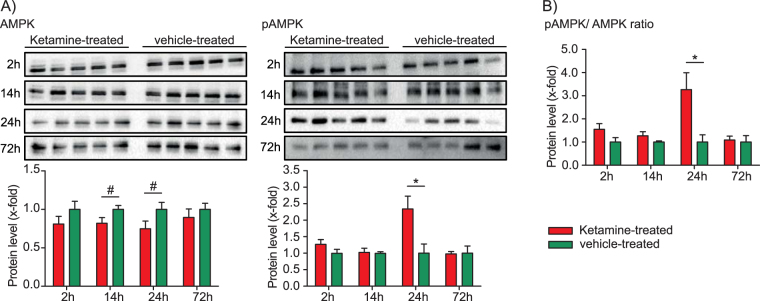
Time-dependent Western blot analysis of AMP-activated protein kinase (AMPK) and phosphorylated AMPK (pAMPK) levels and pAMPK/AMPK ratios reflecting enzyme activation. The results from ketamine-treated animals are normalised to the ones obtained from vehicle treatment (mean value = 1). N = 5 mice per group and time point. ^#^p ≤ 0.10, *p ≤ 0.05. P-values were determined by Student’s t-test. Error bars represent s.e.m.

Based on the above results we next wanted to investigate whether AMP-activated protein Kinase (AMPK) is affected by ketamine treatment. AMPK is the major energy status sensor that promotes catabolic pathways that generate ATP and inhibits anabolic pathways that consume ATP. A low ATP/ADP ratio activates AMPK resulting in its phosphorylation, leads to decreased activities of pathways that consume energy (anabolic pathways) and increased activities of pathways that produce energy (catabolic pathways)^30–36^. Previous data from a study in rats suggest that ketamine’s antidepressant-like effects are mediated by an increased anabolic rate, thereby inducing cell growth and differentiation^26^. The result of an elevated anabolism is a higher energy demand. This is supported by our results of an increased pAMPK/AMPK ratio and decreased ATP/ADP ratio and decreased ATP levels at the 24 h time point^28^. While total AMPK protein levels are not significantly changed, the phosphorylated form of the protein, pAMPK, shows a statistically significant increase at the 24 h time point (Fig. 2, Supplementary Figure 1A and B).

We next investigated whether the observed energy status alterations upon ketamine treatment are associated with changes in the expression of proteins related to cellular energy metabolism. Time-dependent quantitative proteomics profiling analyses were performed 2 h and 24 h after a single injection of a low dose of ketamine. Five mice per time point and treatment group were chosen and MF and CF from ketamine- and vehicle-treated mice were isolated from the hippocampus and quantified using ^15^N metabolically labelled reference proteins by LC-MS/MS analyses.

**Figure 3:**
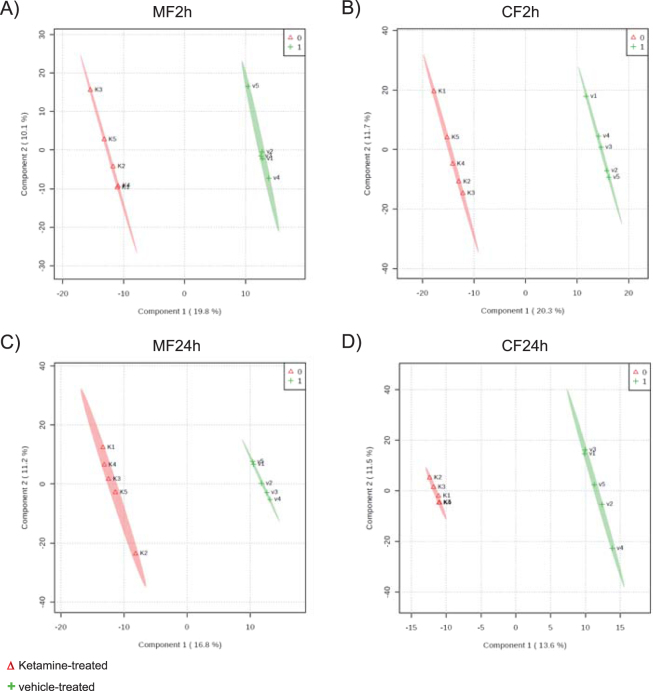
Multivariate partial least squares-discriminant analysis (PLS-DA) of (**A**) membrane-associated (MF2h) and (**B**) cytoplasmic (CF2h) proteins at the 2 h time point and (**C**) membrane-associated (MF24h) and (**D**) cytoplasmic (CF24h) proteins 24 h after a single injection of a low dose of ketamine (3 mg kg^−1^) or vehicle using all quantified proteins. N = 5 mice per group and time point.

**Figure 4:**
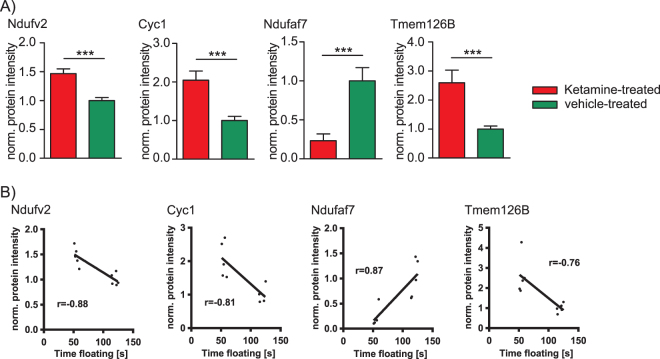
(**A**) Hippocampal OXPHOS protein levels 2 h after a single injection of a low dose of ketamine (3 mg kg^−1^). Complex I NADH dehydrogenase [ubiquinone] flavoprotein 2 (Ndufv2), cytochrome c1, heme protein (Cyc1), and complex I assembly factor (Tmem126B) levels were determined by quantitative proteomics profiling. (**B**) Correlation analyses of Ndufv2, Cyc1, Ndufaf7, and Tmem126B protein levels with forced swim test (FST) floating time 2 h after a single injection of ketamine (p ≤ 0.05). The results from ketamine-treated animals are normalised to the ones obtained from vehicle treatment (mean value = 1). N = 5 mice per group and time point, ***p ≤ 0.001. P-values were determined by Student’s t-test and significance analysis of microarrays and other omic-datasets (SAM). Error bars represent s.e.m. The correlation coefficient r was calculated by Pearson. The linear regression line is only shown for significant (p ≤ 0.05) correlation coefficients.

After proteomics data processing and missing value imputation by k-nearest neighbor (KNN), a total of 889 hippocampal MF and 1173 CF proteins were processed by statistical analyses for the 2 h time point. For the 24 h time point 929 MF and 1091 CF proteins were used for further statistical analyses (Supplemental Table S1). The protein profiles allowed a separation of ketamine- and vehicle-treated mice using multivariate PLS-DA for both time points (Fig. 3). The quality criteria of PLS-DA models were assessed by accuracy, R^2^, and Q^2^ values. They indicate good models for the 2 h time point for MF and CF proteins of ketamine- compared to vehicle-treated animals. The PLS-DA models were only weak for the 24 h time point, which might result in false-positives (Supplemental Table S2). To increase the robustness of the statistical analyses we combined the VIP-score of the PLS-DA with SAM analyses in order to obtain a reliable list of MF and CF proteins that significantly contribute to ketamine’s antidepressant effect. This resulted in 61 MF and 95 CF proteins that were statistically significant altered at the 2 h time point when comparing ketamine- to vehicle-treated mice (Supplemental Table S3). Consistent with previous analyses by us we found proteins that are part of major energy metabolism pathways with altered levels after ketamine treatment^28^. The most significant alterations were detected already 2 h after a single injection of ketamine, in line with the rapid antidepressant effect of ketamine. Subsequent pathway enrichment analyses highlighted altered OXPHOS, ‘purine metabolism’, ‘fructose and mannose metabolism’, ‘proteasome’, ‘protein processing in endoplasmic reticulum’ and ‘ribosome’ **(**Table 1 **)**. We found that already at the 2 h time point a low dose of ketamine resulted in increased levels of several OXPHOS proteins including complex I NADH dehydrogenase [ubiquinone] flavoprotein 2 (Ndufv2), cytochrome c1, heme protein (Cyc1), and complex I assembly factor (Tmem126B) and decreased levels of NADH dehydrogenase [ubiquinone] complex I and assembly factor 7 (Ndufaf7) (Supplemental Table S3 and Fig. 4A). Furthermore, Ndufv2, Cyc1, Ndufaf7 and Tmem126B protein levels significantly correlated with floating time in the FST (Fig. 4B). These results are consistent with our previous metabolomics profiling study that had also indicated mitochondrial energy metabolism, specifically OXPHOS, to be affected by ketamine treatment^28^.

**Table 1:** Pathway enrichment analysis of significantly altered hippocampal proteins (PLS-DA, variable influence of projection (VIP)-score 1.0, significance analysis of microarrays and other omic-datasets (SAM), false-discovery rate (FDR) 0.10, and SAM q 0.10) 2 h after a single injection of a low dose of ketamine (3 mg kg^−1^). N = 5 mice per group and time point.

Pathway	Number of proteins	p-value	FDR
Protein processing in endoplasmic reticulum	6	0,001	0,084
Purine metabolism	6	0,001	0,084
Ribosome	5	0,002	0,084
Fructose and mannose metabolism	3	0,002	0,084
Oxidative phosphorylation (OXPHOS)	5	0,002	0,084
Proteasome	3	0,003	0,100

**Figure 5:**
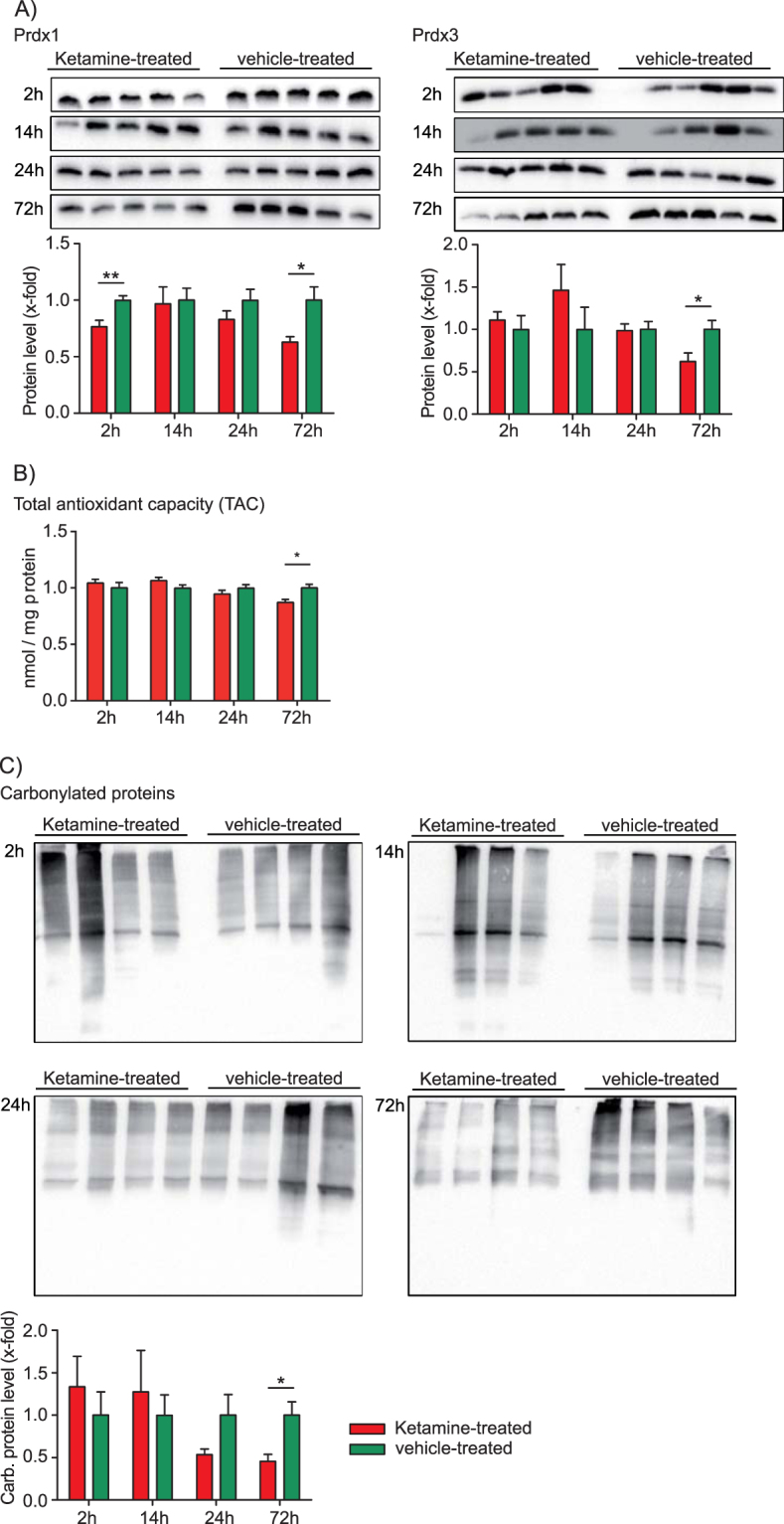
Hippocampal analyses of the antioxidant defense system after a single injection of a low dose of ketamine (3 mg kg^−1^). (**A**) Time-dependent Western blot analysis of peroxiredoxin 1 (Prdx1) and peroxiredoxin 3 (Prdx3). N = 5 mice per group and time point. (**B**) Time-dependent total antioxidant capacity (TAC) analysis. N = 5 mice per group and time point. (**C**) OxyBlot analysis of the time-dependent protein damage by protein carbonylation in the hippocampus after ketamine treatment. N = 4 mice per group and time point. The results from ketamine-treated animals are normalised to the ones obtained from vehicle treatment (mean value = 1). *p ≤ 0.05, **p ≤ 0.01. P-values were determined by Student’s t-test. Error bars represent s.e.m.

Increased OXPHOS activity can result in higher ROS levels. The phenomenon referred to as oxidative stress causes an imbalance of ROS and the cellular defense system made up of antioxidant molecules and enzymes like peroxiredoxins (Prdxs) that contribute to the antioxidant capacity of the cell^37–41^. We therefore next analysed ketamine’s effects on oxidative stress. Cytoplasmic Prdx1 and mitochondrial Prdx3 protein levels were significantly lower at the 2 h and 72 h time points, respectively (Fig. 5A, Supplementary Figure 1C). In addition, we found decreased total antioxidant capacity (TAC) 72 h after ketamine injection (Fig. 5B, supplementary Figure 1D). ROS radicals promoting oxidative stress result in cellular damage by reacting with deoxyribonucleic acid (DNA), ribonucleic acid (RNA), lipids, and proteins. Protein modifications by ROS become apparent through the introduction of carbonyl groups into protein side chains. Since ROS are difficult to measure due to their highly reactive nature we assessed their presence indirectly by the analysis of carbonylated (damaged) proteins^37^. Our results indicate that ketamine results in statistically significant reduction of protein carbonylation 72 h after ketamine treatment (Fig. 5C).

## Discussion

Ketamine treatment reduced the ATP/ADP metabolite ratio at 2 h and 24 h and both ATP and ADP levels significantly correlated with FST floating time at 24 h suggesting an energy deficit. This is consistent with our previous results where we found a significant reduction of ATP levels at 24 h^28^. Also in line with this is our observation of an increased AMPK activation following ketamine treatment. In the current study we observed several metabolite level and metabolite ratio changes that are part of the ‘citrate cycle’ and ‘glycolysis’ as early as 2 h after ketamine administration, reflecting the fast antidepressant effect of the drug. The ‘citrate cycle’ is connected to the OXPHOS pathway which produces energy in the form of ATP. ‘Glycolysis’ is a major catabolic cytoplasmic pathway where glucose is degraded to pyruvate, which is further metabolized by pyruvate dehydrogenase complex to acetyl-CoA that enters the ‘citrate cycle’. The latter is a mitochondrial matrix pathway that is coupled to the OXPHOS pathway^29^. We observed metabolite ratio changes that are either due to protein level or protein activity alterations of OXPHOS complex II enzymatic subunit A that processes these metabolites^28,42^. Our previous time-dependent hippocampal metabolomics profiling analyses indicated statistically significant biomolecular changes that are part of several pathways including the ‘citrate cycle’ and ‘glycolysis’^28^. Mitochondrial abnormalities including alterations in energy metabolism have been implicated in the pathobiology of affective disorders^43–45^. Taken together, these findings provide further evidence for an important role of mitochondrial energy metabolism in ketamine’s mode of action to generate ATP which compensates for the increased energy demand at activated synapses. We propose that ketamine treatment causes a cellular energy deficit by first promoting anabolic processes (that consume ATP) through the activation of mTORC1. This is counterbalanced in a second phase by the activation of AMPK that promotes catabolic pathways (to generate ATP).

Ketamine affects glutamate neurotransmission and is tightly linked to mitochondrial energy metabolism. Upon release, glutamate is taken up by glial cells and subsequently metabolized to glutamine. In addition, glutamate is metabolized from α-ketoglutarate, a ‘citrate cycle’ metabolite, and can also be recycled through this pathway. Interestingly, glutamate neurotransmission also stimulates glucose utilization and ‘glycolysis’, the upstream pathway of the OXPHOS pathway^46^. Magnetic resonance spectroscopy analysis of rat medial prefrontal cortex with ^13^C-labeled glucose demonstrated ^13^C-enrichment of glutamate, glutamine and GABA upon ketamine treatment, reflecting neurotransmitter release coupled to glycolytic and ‘citrate cycle’ activity. In addition, other studies have shown elevated medial prefrontal cortex metabolism and an involvement of mitochondria in MDD pathobiology^43,47–49^. Mitochondrial respiration in peripheral blood mononuclear cells from depressed patients is lower compared to cells from control subjects and correlates with depressive symptom severity^50^. Furthermore, mitochondrial disorders are often comorbid with psychiatric disorders including MDD and patients frequently suffer from TRD. These patients have a primary impairment of mitochondrial functioning through either nuclear or mitochondrial DNA mutations. Mitochondrial impairment mostly affects high energy consuming organs like muscles and brain. The mitochondrial disorder is often recognized only later in life of MDD patients^51–53^. Previous studies have connected the antidepressant response with drugs elevating ATP levels. Spectroscopy data have shown decreased NPT levels (mainly ATP) in the brain of depressed patients. Another magnetic resonance spectroscopy study could demonstrate lower NPT levels (mainly ATP) in fluoxetine responders compared with non-responders. Previous data from a study in rats have suggested that the antidepressant-like effect observed after a single injection of ketamine is mediated by an increased anabolic rate-mediating cell growth and differentiation, processes that are highly energy consuming^54,55^. The high ketamine treatment efficacy for TRD patients might be due to the drug’s beneficial effects on mitochondrial energy metabolism. We submit that mitochondria play a critical role in the development of MDD as well as AD treatment response, at least in a subset of patients with a defined symptomatology.

In order to better understand the biomolecular pathways and cellular mechanisms underlying ketamine’s mode of action, a time-dependent proteomics profiling analysis was also performed. We identified several pathways that were affected and associated with mitochondrial energy metabolism. The PLS-DA showed a time-dependent separation of ketamine- and vehicle-treated mice that was evident by significant protein level alterations and overrepresented pathways. Of the pathways that we found to be affected by ketamine treatment we focused our attention on OXPHOS, in line with results previously obtained by us that had implicated mitochondrial energy metabolism to play a major role in the drug’s mode of action. Ketamine affects the OXPHOS pathway as is evident by several complex I protein levels (Ndufv2, Cyc1, Ndufaf7 and Tmem126B) that correlate with FST floating time in a treatment time-dependent manner.

Taken together, we propose a mechanism by which ketamine stimulates glutamatergic neurotransmission and neuronal activity, ultimately resulting in a long-term potentiation (LTP)-like process. This activation results in the activation of mTORC1. The mTORC1 mediated anabolic processes include protein synthesis and mitochondrial biogenesis within 30 minutes and 1 h upon ketamine treatment. Pretreatment with rapamycin, an mTOR inhibitor, prevents ketamine’s antidepressant-like effect^26^. Furthermore, other studies indicated that mTOR inhibition decreases mitochondrial respiration, whereas mTOR hyperactivation elevates the expression of oxidative metabolism genes and mitochondrial DNA copy number^56–58^. Hence, ketamine may not only increase protein translation through mTORC1, but also mitochondrial biogenesis. LTP-like processes require energy in the form of ATP followed by AMPK activation, which we found in the present study, in order to promote catabolic processes to generate ATP. Ketamine’s antidepressant-like effects are known to last for one to two weeks. This long-term effect can be explained by the observed LTP-like process. Late phase LTP with spine formation and strengthening of synaptic connections are known to last from days to weeks. Increased OXPHOS activity can result in elevated ROS levels, a phenomenon referred to as oxidative stress, which causes protein, DNA, RNA and lipid modifications and damage. We found that protein carbonylation decreases over time until it reaches significance at the 72 h time point. This can be explained by either decreased ROS levels or alternatively by an activation of a cellular defense system that captures ROS. The cellular defense system includes antioxidant molecules and enzymes such as Prdxs that combat ROS. In addition, a protein quality control system that degrades damaged proteins counteracts oxidative stress^38–41,59,60^. We found that total antioxidant capacity (TAC) and cytoplasmic Prdx1 levels were lower at the 2 h and 72 h time points and mitochondrial Prdx3 levels lower at the 72 h time point in ketamine- vs. vehicle-treated mice. The reduced protein carbonylation we observed in ketamine- vs. vehicle-treated mice could be indicative of increased protein quality control processes in the cell. This is supported by our proteomic data that indicate an upregulation of the ‘proteasome’ pathway at the 2 h time point. Taken together, our combined proteomic results suggest that ketamine administration in mice leads to increased degradation of damaged proteins and homeostatic redox adjustment.

The close connection between the glutamatergic system and mitochondrial energy metabolism make a compelling case for the here observed facilitative effects of energy metabolism and the antioxidant defense system following ketamine administration. Further experiments including blocking pathway enzymes and receptors including OXPHOS complex I and AMPK that we found to be involved as well as different ketamine doses are required to confirm these findings. We only assessed ketamine’s behavioral effect using FST floating time and did not carry out any additional behavioral analyses like the learned helplessness, chronic mild stress and novelty suppressed feeding tests that have been previously reported by others. However, using several consecutive behavioral stress assays will in all likelihood affect the animals’ metabolome and proteome and therefore skew the data.

## Methods

### Animals and ketamine treatment

The animals and ketamine treatment were housed and carried out as previously described^28^. The experiments were performed in accordance with European Communities Council Directive 86/609/EEC. The protocols were approved by the committee for the Care and Use of Laboratory Animals of the Government of Upper Bavaria, Germany.

### Forced swim test

The forced swim test and floating time analysis were carried out as previously described^28^.

### Isolation of cytoplasmic protein fraction (CF) and membrane-associated protein fraction (MF)

CF and MF were prepared by repeated tissue homogenization and extraction of non-MF proteins and solubilization of MF proteins with sodium dodecyl sulfate (SDS). Tissues were homogenized for 30 s in 2 M NaCl, 10 mM Hepes/NaOH, pH 7.4, containing 1 mM ethylenediaminetetraacetic acid (EDTA), phosphatase inhibitor cocktail 2 and 3 (Sigma-Aldrich, Munich, Germany), protease inhibitor cocktail tablets ‘cOmplete’ (Roche Diagnostics, Mannheim, Germany), incubated for 10 min and homogenized for 30 s and further with a ultrasonicator for 3 × 10 s on ice. Homogenates were centrifuged at 16100 g at 4 °C for 20 min with the supernatant containing CF proteins. Pellets were rehomogenized in 0.1 M Na_2_CO_3_ and 1 mM EDTA containing 1 mM EDTA, phosphatase inhibitor cocktail 2 and 3 (Sigma-Aldrich, Munich, Germany), protease inhibitor cocktail tablets ‘cOmplete’ (Roche Diagnostics, Mannheim, Germany), pH 11.3, mixed at 4 °C for 30 min and collected by centrifugation (16100 g at 4 °C for 20 min). Subsequently, pellets were extracted with 5 M urea, 100 mM NaCl, 10 mM Hepes, pH 7.4 and 1 mM EDTA containing 1 mM EDTA, phosphatase inhibitor cocktail 2 and 3 (Sigma-Aldrich, Munich, Germany), protease inhibitor cocktail tablets ‘cOmplete’ (Roche Diagnostics, Mannheim, Germany) and then washed twice with 0.1 M Tris-HCl, containing 1 mM EDTA, phosphatase inhibitor cocktail 2 and 3 (Sigma-Aldrich, Munich, Germany), protease inhibitor cocktail tablets ‘cOmplete’ (Roche Diagnostics, Mannheim, Germany) pH 7.6. Pellets were solubilized in 2% SDS, 50 mM dithiothreitol (DTT) and 0.1 M Tris-HCl, containing 1 mM EDTA, phosphatase inhibitor cocktail 2 and 3 (Sigma-Aldrich, Munich, Germany), protease inhibitor cocktail tablets ‘cOmplete’ (Roche Diagnostics, Mannheim, Germany) pH 7.6, at 90 °C for 1 min and stored at −20 °C until further analysis.

### Western blot

Hippocampal MF and CF proteins from 8-week-old male C57BL/6 mice treated with ketamine for 2 h, 14 h, 24 h and 72 h were fractionated by SDS-polyacrylamide gel electrophoresis (SDS-PAGE) and Western blot was performed based on standard protocols. After electrophoresis, proteins were transferred to PVDF membranes (Immobilon-P, Millipore, Billerica, USA). Primary antibodies were against adenosinmonophosphate-activated protein kinase (AMPK, Abcam, Cambridge, UK), phosphorylated AMPK (pAMPK, Cell Signaling, Merck, Darmstadt, Germany), peroxiredoxin 1 (Prdx1, Abcam, Cambridge, UK), peroxiredoxin 3 (Prdx3, Abcam, Cambridge, UK). Anti-rabbit, anti-mouse and anti-goat ECL horseradish peroxidase-linked secondary antibodies (GE Healthcare Life Sciences, Little Chalfont, Buckinghamshire, UK) were used. The densitometric analyses were performed with the Image Lab software (Bio-Rad, Munich, Germany).

### Protein oxidation analysis

The OxyBlot^TM^ Kit (Merck Millipore, Darmstadt, Germany) was used according to the manufacturer’s instructions.

### Total antioxidant capacity (TAC)

The TAC Assay Kit (BioVision, Milpitas, USA) was used according to the manufacturer’s instructions. Absorbance was measured at 570 nm using a plate reader (iMark^Tm^ Microplate Absorbance Reader, Bio-Rad, Munich, Germany).

### Protein sample preparation for quantitative proteomics analyses

Hippocampal CF and MF proteins were mixed 1:1 with CF and MF ^15^N-labeled protein standards, respectively. The latter were derived from C57BL/6 mice that had been fed a ^15^N-labelled diet (Silantes, Munich, Germany) for 12 weeks. 50 μg of the ^14^N/^15^N protein mixture were separated by SDS-PAGE. Separated proteins were stained with Coomassie Brilliant Blue for 20 min and destained overnight. Each gel lane was cut into 16 2.5 mm slices per biological replicate and each slice further cut into smaller pieces.

The gel pieces were then covered with 100 µl of 25 mM NH_4_HCO_3_/50% acetonitrile (ACN) for complete destaining and mixed for 10 min at room temperature. The supernatant was discarded and this step repeated twice. Proteins were reduced with 75mL 1x DTT/25 mM NH_4_HCO_3_ and incubated at 56 °C for 30 min in the dark. The supernatant was discarded and for alkylation 100 µl iodoacetamide (IAM) was added to the gel pieces and mixed for 30 min at room temperature. The supernatant was discarded and the gel pieces washed twice with 100 µl 25 mM Na_4_HCO_3_/50% ACN and incubated for 10 min at room temperature. The supernatant was discarded and gel pieces dried for 20 min at room temperature. Proteins were digested with 50 µl trypsin solution (5ng/µl trypsin/25 mM NH_4_HCO_3_) overnight at 37 °C. Peptides were extracted from the gel pieces by incubation in 50 µl 2% formic acid (FA)/50% ACN for 20 min at 37 °C followed by 5 min sonication. This step was repeated twice with 50 µL of 1% FA/50% ACN. The supernatants were then combined and dried (SpeedVac Plus, SC 210 A, Savant, Hyannis, MA, USA). The pellet was stored at −20 °C.

### Liquid chromatography tandem mass spectrometry (LC-MS/MS)

Hippocampal MF and CF proteins were identified and quantified with a Dionex Ultimate 3000 RSLC nanoUPLC (Thermo Fisher Scientific, Waltham, USA) online coupled to a QExactive^TM^ Orbitrap^TM^ mass spectrometer (Thermo Fisher Scientific, Waltham, USA). Separation of peptides was performed by reversed-phase chromatography at a flow rate of 300 nl/min (Thermo Scientific PepMap C18, 2 µm particle size, 100 A pore size, 75 µm × 50 cm). Peptides were loaded onto a pre-column (Thermo Scientific PepMap 100 C18. 5 µm particle size, 100 A pore size, 300 µm × 5 mm) in 0.1% FA for 3 min at a flow rate of 10 µl/min. Chromatography was performed using the following solvents: solvent A water, 0.1% FA, solvent B 80% ACN, 20% water, 0.1% FA. A linear gradient of 2–40% B for 30 min was used. The LC eluant was sprayed into the mass spectrometer by means of an easy-spray source (Thermo Fisher Scientific, Waltham, USA). All *m/z* values of eluting ions were measured in the Orbitrap mass analyzer, set at a resolution of 70000. Data dependent scans (Top 20) were employed to automatically isolate and generate fragment ions by high energy collisional dissociation in the quadrupole mass analyser and measurement of the resulting fragment ions was performed in the Orbitrap analyzer, set at a resolution of 17500. Peptide ions with charge states of 2+ to 4+ and above were selected for fragmentation.

Orbitrap raw files were converted to mzXML files using MSConvert software. The in-house software package iSPY, which was adapted from an earlier version of a peptide quantitation program known as iTracker, was used to identify and quantify peptides^61,62^. The software was used to convert mzXML to mgf files that were then imported into Mascot and searched against the SwissProt Mouse database (November 2013) and a decoy database. The search was then performed using TAXONOMY = Mus. The databases were searched using the following settings: variable modifications of carbamidomethyl, oxidation), 20 ppm peptide tolerance, 0.1 Da MS/MS tolerance, 2 missed cleavages and peptide charge states of +2, +3, or +4. In iSPY, Mascot dat output files were run through Percolator for improved identification. Non-unique peptides were discarded. Only peptides with a protein type 1 error of less than 0.01 were kept in the final dataset. The heavy and light peak intensities for each peptide were calculated in iSPY using retention time and sequence information from the MS1 spectra and Mascot search, respectively. Briefly, the intensities for a pre-specified number of isotopomeric peaks were calculated by scanning through a retention time window spanning a set distance on either side of the maximum intensity value. The ^14^N and ^15^N peptide isotopic peaks from the MS1 dataset were used to compare the theoretical mass difference between the heavy and light peptides, and the typical isotopic distribution patterns. Only quantifiable peptides for which both heavy and a light peak intensities were identified in five replicates were included in the dataset.

### Isolation of polar metabolites from mouse tissue and metabolomics analyses

Samples were obtained, measured and analysed as previously described^28,63^.

### Statistics and data analyses

#### Identification of significant metabolite and protein level alterations

Metabolite intensities as well as protein ratios were median-normalised and auto-scaled for statistical analysis. Significant protein level changes 2 h and 24 h after ketamine treatment were identified by multivariate partial least squares-discriminant analyses (PLS-DA) and high-dimensional feature selection significance analysis of microarrays and other omic-datasets (SAM) using MetaboAnalyst^64,65^. The quality of the PLS-DA models were assessed for R^2^, Q^2^ and accuracy values with variable influence of projection (VIP)-score ≥ 1.0 and for SAM with q ≤ 0.10 and false discovery rate (FDR) ≤ 0.10^66^. We improved robustness of our data analyses and increased confidence in significantly altered metabolites and proteins as well as in the subsequent analyses of overrepresented KEGG pathways by only considering the overlap between the two different statistical methods.

#### Identification of significantly enriched pathways

Pathway analyses were performed using MetaboAnalyst^64^ applying a hypergeometric algorithm for overrepresentation analysis and relative-betweeness centrality for pathway topology analysis. Pathways were considered affected if they were significantly enriched for all significantly altered metabolites (P_Holm-corrected_ ≤ 0.05). Proteomic pathway enrichment was assessed by String with an FDR ≤ 0.10^67,68^.

#### Calculation of metabolite pair ratios

The metabolite pair ratios were calculated as described^28^.

All data generated or analysed during this study are included in this published article (and its Supplementary Information files).

## Electronic supplementary material

Supplementary Table 1

Supplementary Table 2

Supplementary Table 3

Supplementary Figure 1

## References

[1] Olesen J (2006). Consensus document on European brain research. J Neurol Neurosurg Psychiatry.

[2] Murray CJ, Lopez AD (1997). Alternative projections of mortality and disability by cause 1990-2020: Global Burden of Disease Study. Lancet.

[3] Agid Y (2007). How can drug discovery for psychiatric disorders be improved?. Nat Rev Drug Discov.

[4] Kessler RC (2009). The global burden of mental disorders: an update from the WHO World Mental Health (WMH) surveys. Epidemiol Psichiatr Soc.

[5] Kessler RC (2003). Epidemiology of women and depression. J Affect Disord.

[6] Organization, W. H. Depression. (2016).

[7] Trivedi MH (2006). Evaluation of outcomes with citalopram for depression using measurement-based care in STAR*D: implications for clinical practice. Am J Psychiatry.

[8] Sonnenberg CM, Deeg DJ, Comijs HC, van Tilburg W, Beekman AT (2008). Trends in antidepressant use in the older population: results from the LASA-study over a period of 10 years. J Affect Disord.

[9] Racagni G, Popoli M (2008). Cellular and molecular mechanisms in the long-term action of antidepressants. Dialogues Clin Neurosci.

[10] Kessler RC (2003). The epidemiology of major depressive disorder: results from the National Comorbidity Survey Replication (NCS-R). JAMA.

[11] Berman RM (2000). Antidepressant effects of ketamine in depressed patients. Biol Psychiatry.

[12] Hirota K, Lambert DG (1996). Ketamine: its mechanism(s) of action and unusual clinical uses. Br J Anaesth.

[13] Krystal JH (1999). NMDA agonists and antagonists as probes of glutamatergic dysfunction and pharmacotherapies in neuropsychiatric disorders. Harv Rev Psychiatry.

[14] Krystal JH (2002). Glutamate and GABA systems as targets for novel antidepressant and mood-stabilizing treatments. Mol Psychiatry.

[15] Krystal JH, Sanacora G, Duman RS (2013). Rapid-acting glutamatergic antidepressants: the path to ketamine and beyond. Biol Psychiatry.

[16] Kashiwagi K (2002). Channel blockers acting at N-methyl-D-aspartate receptors: differential effects of mutations in the vestibule and ion channel pore. Mol Pharmacol.

[17] Kotermanski SE, Johnson JW (2009). Mg2+ imparts NMDA receptor subtype selectivity to the Alzheimer’s drug memantine. J Neurosci.

[18] aan het Rot M (2010). Safety and efficacy of repeated-dose intravenous ketamine for treatment-resistant depression. Biol Psychiatry.

[19] Diazgranados N (2010). A randomized add-on trial of an N-methyl-D-aspartate antagonist in treatment-resistant bipolar depression. Arch Gen Psychiatry.

[20] Larkin GL, Beautrais AL (2011). A preliminary naturalistic study of low-dose ketamine for depression and suicide ideation in the emergency department. Int J Neuropsychopharmacol.

[21] Chatterjee M, Ganguly S, Srivastava M, Palit G (2011). Effect of ‘chronic’ versus ‘acute’ ketamine administration and its ‘withdrawal’ effect on behavioural alterations in mice: implications for experimental psychosis. Behav Brain Res.

[22] Chatterjee M, Verma R, Ganguly S, Palit G (2012). Neurochemical and molecular characterization of ketamine-induced experimental psychosis model in mice. Neuropharmacology.

[23] Swerdlow NR, Taaid N, Oostwegel JL, Randolph E, Geyer MA (1998). Towards a cross-species pharmacology of sensorimotor gating: effects of amantadine, bromocriptine, pergolide and ropinirole on prepulse inhibition of acoustic startle in rats. Behav Pharmacol.

[24] Cilia J, Hatcher P, Reavill C, Jones DN (2007). (+/−) Ketamine-induced prepulse inhibition deficits of an acoustic startle response in rats are not reversed by antipsychotics. J Psychopharmacol.

[25] Javitt DC, Zukin SR (1991). Recent advances in the phencyclidine model of schizophrenia. Am J Psychiatry.

[26] Li N (2010). mTOR-dependent synapse formation underlies the rapid antidepressant effects of NMDA antagonists. Science.

[27] Zanos P (2016). NMDAR inhibition-independent antidepressant actions of ketamine metabolites. Nature.

[28] Weckmann K, Labermaier C, Asara JM, Müller MB, Turck CW (2014). Time-dependent metabolomic profiling of Ketamine drug action reveals hippocampal pathway alterations and biomarker candidates. Transl Psychiatry.

[29] Jeremy M. Berg, J. L. T. and Lubert Stryer. *Biochemistry*. *5th edition*. (2002).

[30] Amodeo GA, Rudolph MJ, Tong L (2007). Crystal structure of the heterotrimer core of Saccharomyces cerevisiae AMPK homologue SNF1. Nature.

[31] Corton JM, Gillespie JG, Hawley SA, Hardie DG (1995). 5-aminoimidazole-4-carboxamide ribonucleoside. A specific method for activating AMP-activated protein kinase in intact cells?. Eur J Biochem.

[32] Townley R, Shapiro L (2007). Crystal structures of the adenylate sensor from fission yeast AMP-activated protein kinase. Science.

[33] Xiao B (2007). Structural basis for AMP binding to mammalian AMP-activated protein kinase. Nature.

[34] Xiao B (2011). Structure of mammalian AMPK and its regulation by ADP. Nature.

[35] Xiao B (2013). Structural basis of AMPK regulation by small molecule activators. Nat Commun.

[36] Barnes K (2002). Activation of GLUT1 by metabolic and osmotic stress: potential involvement of AMP-activated protein kinase (AMPK). J Cell Sci.

[37] Lushchak VI (2015). Free Radicals, Reactive Oxygen Species, Oxidative Stresses And Their Classifications. Ukr Biochem J.

[38] Ruszkiewicz J, Albrecht J (2015). Changes in the mitochondrial antioxidant systems in neurodegenerative diseases and acute brain disorders. Neurochem Int.

[39] Begara-Morales JC (2016). Antioxidant Systems are Regulated by Nitric Oxide-Mediated Post-translational Modifications (NO-PTMs). Front Plant Sci.

[40] Wang, X. & Hai, C. Novel insights into redox system and the mechanism of redox regulation. *Mol Biol Rep*, 10.1007/s11033-016-4022-y (2016). 10.1007/s11033-016-4022-y 27255468

[41] Rhee SG (2016). Overview on Peroxiredoxin. Mol Cells.

[42] Petersen AK (2012). On the hypothesis-free testing of metabolite ratios in genome-wide and metabolome-wide association studies. BMC Bioinformatics.

[43] Webhofer C (2013). Proteomic and metabolomic profiling reveals time-dependent changes in hippocampal metabolism upon paroxetine treatment and biomarker candidates. J Psychiatr Res.

[44] Filiou MD (2011). Proteomics and metabolomics analysis of a trait anxiety mouse model reveals divergent mitochondrial pathways. Biol Psychiatry.

[45] Scaini G (2010). Evaluation of Krebs cycle enzymes in the brain of rats after chronic administration of antidepressants. Brain Res Bull.

[46] Magistretti PJ, Pellerin L, Rothman DL, Shulman RG (1999). Energy on demand. Science.

[47] Webhofer C (2011). Metabolite profiling of antidepressant drug action reveals novel drug targets beyond monoamine elevation. Transl Psychiatry.

[48] Glancy B, Balaban RS (2012). Role of mitochondrial Ca2+ in the regulation of cellular energetics. Biochemistry.

[49] Chowdhury GM (2017). Transiently increased glutamate cycling in rat PFC is associated with rapid onset of antidepressant-like effects. Mol Psychiatry.

[50] Karabatsiakis A (2014). Mitochondrial respiration in peripheral blood mononuclear cells correlates with depressive subsymptoms and severity of major depression. Transl Psychiatry.

[51] Anglin, R. E., Rosebush, P. I. & Mazurek, M. F. Treating psychiatric illness in patients with mitochondrial disorders. *Psychosomatic*s **51**, 179; author reply 179–180, 10.1176/appi.psy.51.2.179 (2010). 10.1176/appi.psy.51.2.179 20332296

[52] Anglin RE, Garside SL, Tarnopolsky MA, Mazurek MF, Rosebush PI (2012). The psychiatric manifestations of mitochondrial disorders: a case and review of the literature. J Clin Psychiatry.

[53] Anglin RE, Tarnopolsky MA, Mazurek MF, Rosebush PI (2012). The psychiatric presentation of mitochondrial disorders in adults. J Neuropsychiatry Clin Neurosci.

[54] Moore CM, Christensen JD, Lafer B, Fava M, Renshaw PF (1997). Lower levels of nucleoside triphosphate in the basal ganglia of depressed subjects: a phosphorous-31 magnetic resonance spectroscopy study. Am J Psychiatry.

[55] Volz HP (1998). 31P magnetic resonance spectroscopy in the frontal lobe of major depressed patients. Eur Arch Psychiatry Clin Neurosci.

[56] Cunningham JT (2007). mTOR controls mitochondrial oxidative function through a YY1-PGC-1alpha transcriptional complex. Nature.

[57] Morita M (2013). mTORC1 controls mitochondrial activity and biogenesis through 4E-BP-dependent translational regulation. Cell Metab.

[58] Ramanathan A, Schreiber SL (2009). Direct control of mitochondrial function by mTOR. Proc Natl Acad Sci USA.

[59] Coux O, Tanaka K, Goldberg AL (1996). Structure and functions of the 20S and 26S proteasomes. Annu Rev Biochem.

[60] Kastle M, Grune T (2011). Protein oxidative modification in the aging organism and the role of the ubiquitin proteasomal system. Curr Pharm Des.

[61] Gutteridge A (2010). Nutrient control of eukaryote cell growth: a systems biology study in yeast. BMC Biol.

[62] Marondedze C, Groen AJ, Thomas L, Lilley KS, Gehring C (2016). A Quantitative Phosphoproteome Analysis of cGMP-Dependent Cellular Responses in Arabidopsis thaliana. Mol Plant.

[63] Yuan M, Breitkopf SB, Yang X, Asara JM (2012). A positive/negative ion-switching, targeted mass spectrometry-based metabolomics platform for bodily fluids, cells, and fresh and fixed tissue. Nat Protoc.

[64] Xia J, Psychogios N, Young N, Wishart DS (2009). MetaboAnalyst: a web server for metabolomic data analysis and interpretation. Nucleic Acids Res.

[65] Tusher VG, Tibshirani R, Chu G (2001). Significance analysis of microarrays applied to the ionizing radiation response. Proc Natl Acad Sci USA.

[66] Svante W, Michael S, Lennart E (2001). In Chemometrics and Intelligent Laboratory Systems.

[67] Hosack DA, Dennis G, Sherman BT, Lane HC, Lempicki RA (2003). Identifying biological themes within lists of genes with EASE. Genome Biol.

[68] Szklarczyk D (2017). The STRING database in 2017: quality-controlled protein-protein association networks, made broadly accessible. Nucleic Acids Res.

