# Supplementary material for: Ketamine’s antidepressant effect is mediated by energy metabolism and antioxidant defense system
Source: Sci Rep. 2017 Nov 17;7:15788. doi: 10.1038/s41598-017-16183-x (PMC5694011; doi:10.1038/s41598-017-16183-x)
Supplement: Supplementary file 4 — Supplementary Figure 1 [file 41598_2017_16183_MOESM4_ESM.pdf]

Ketamine's antidepressant effect is mediated by energy metabolism and antioxidant defense system

Weckmann Katja[1,+]; Deery Mike[2]; Howard Julie[2]; Feret Renata[2]; Asara John M[3]; Dethloff Frederik[1]; Filiou Michaela D[4]; Iannace Jamie[1]; Labermaier Christiana[1]; Maccarrone Giuseppina[1]; Webhofer Christian[1]; Teplytska Larysa[1]; Lilley Kathryn[2]; Müller Marianne[5]; Turck Christoph W[1*]

[1]*Max Planck Institute of Psychiatry, Department of Translational Research in Psychiatry, Munich, Germany;*

[2]*Cambridge Centre for Proteomics, Cambridge System Biology Centre, University of Cambridge, Cambridge, UK;*

[3]*Division of Signal Transduction, Beth Israel Deaconess Medical Center and Department of Medicine, Harvard Medical School, Boston, USA;*

[4]*Max Planck Institute of Psychiatry, Department of Stress Neurobiology and Neurogenetics, Munich, Germany*

[5]*Experimental Psychiatry, Department of Psychiatry and Psychotherapy & Focus Program Translational Neuroscience, Johannes Gutenberg University Medical Center, Mainz, Germany*

[+]*Present address: Institute of Pathobiochemistry, Johannes Gutenberg University, Medical School, Mainz, Germany*

***Corresponding author**:

Prof. Dr. Christoph W. Turck

Max Planck Institute of Psychiatry, Department of Translational Research in Psychiatry, Kraepelinstrasse 2-10, 80804 Munich, Germany

Phone: +49-89-30622317

E-mail: turck@psych.mpg.de

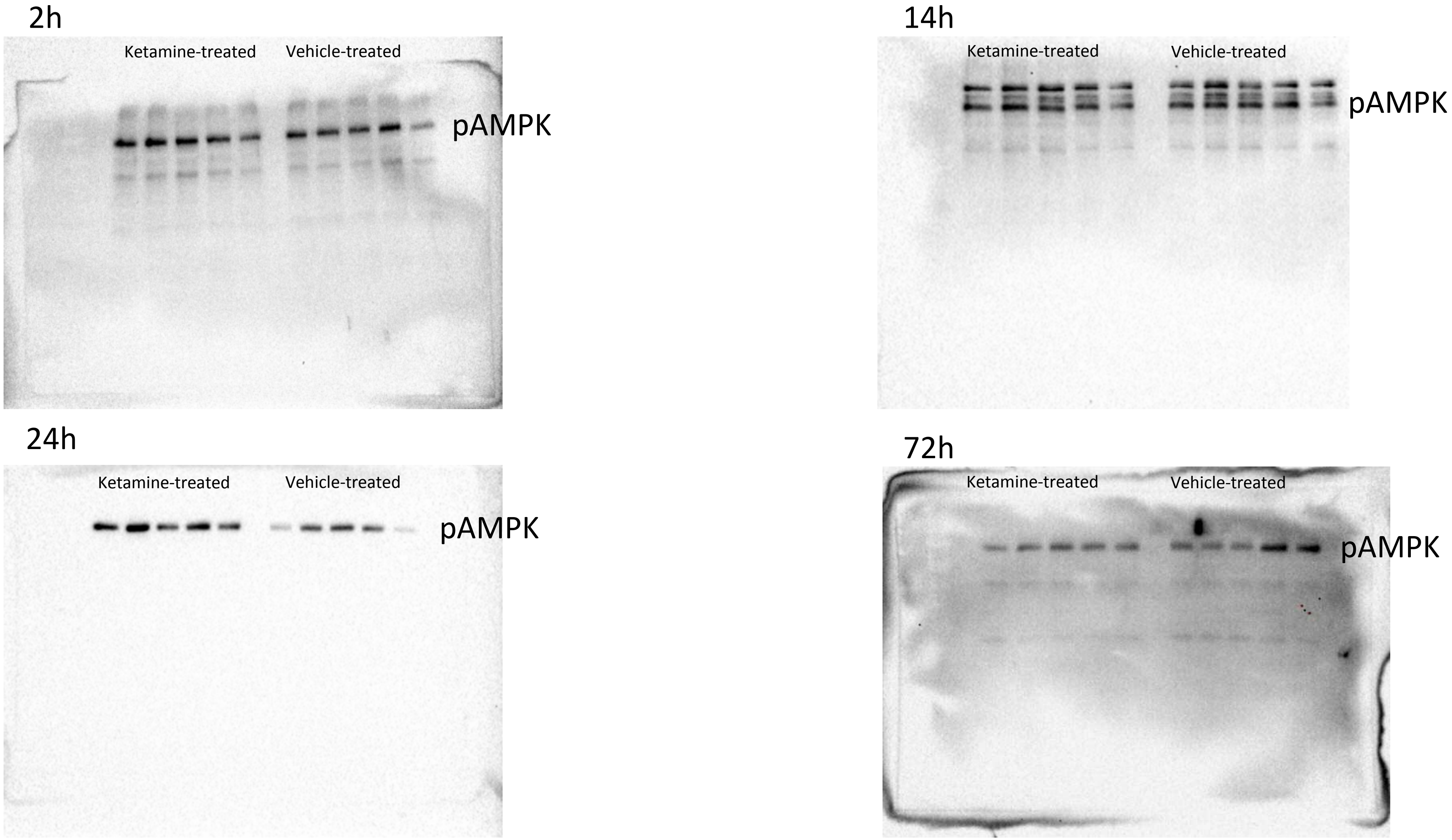

Supplemental figure 1A: Time-dependent Western blot analysis of AMP-activated protein kinase (AMPK). N=5 mice per group and time point.

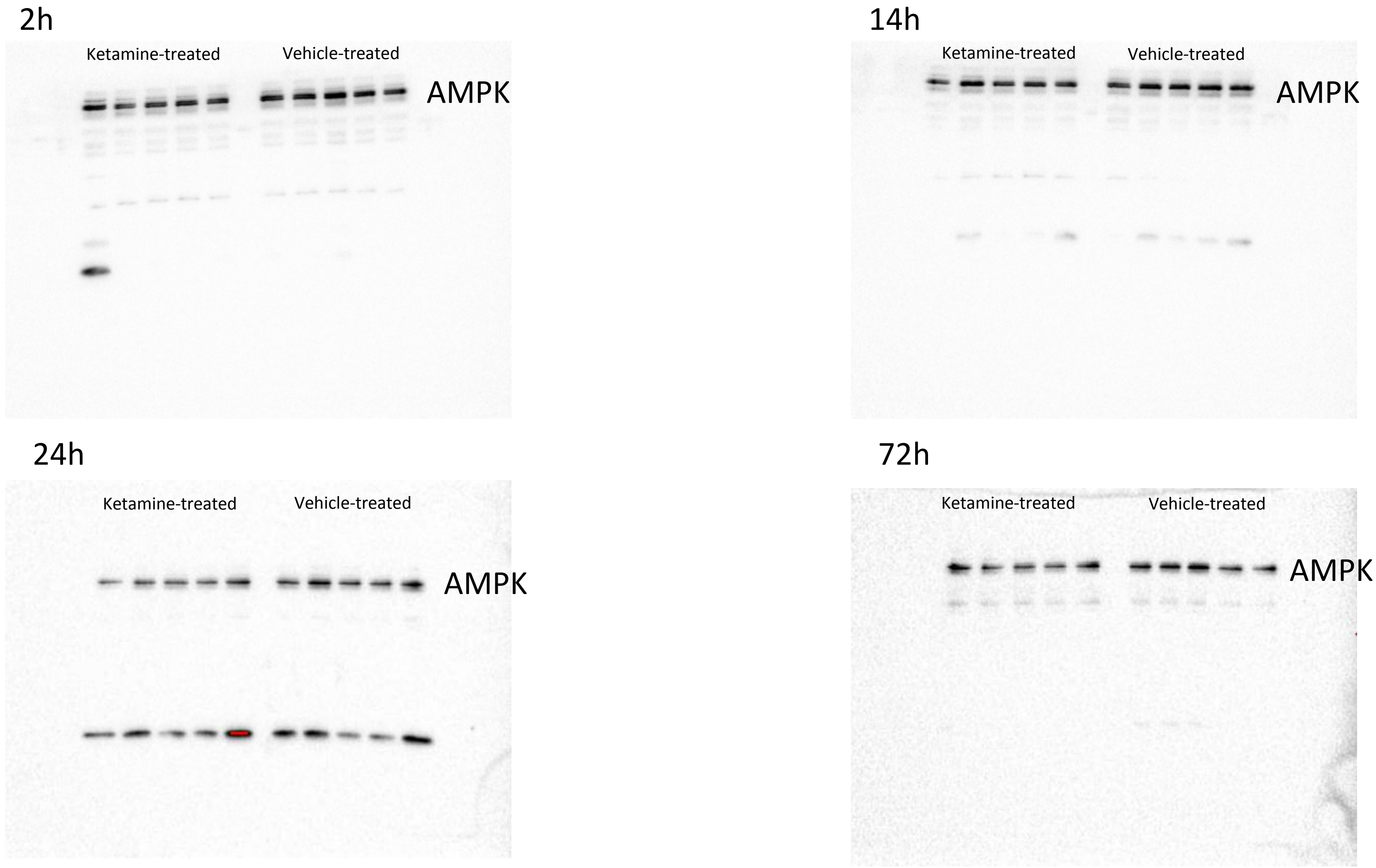

Supplemental figure 1B: Time-dependent Western blot analysis of phosphorylated AMP-activated protein kinase (pAMPK). N=5 mice per group and time point.

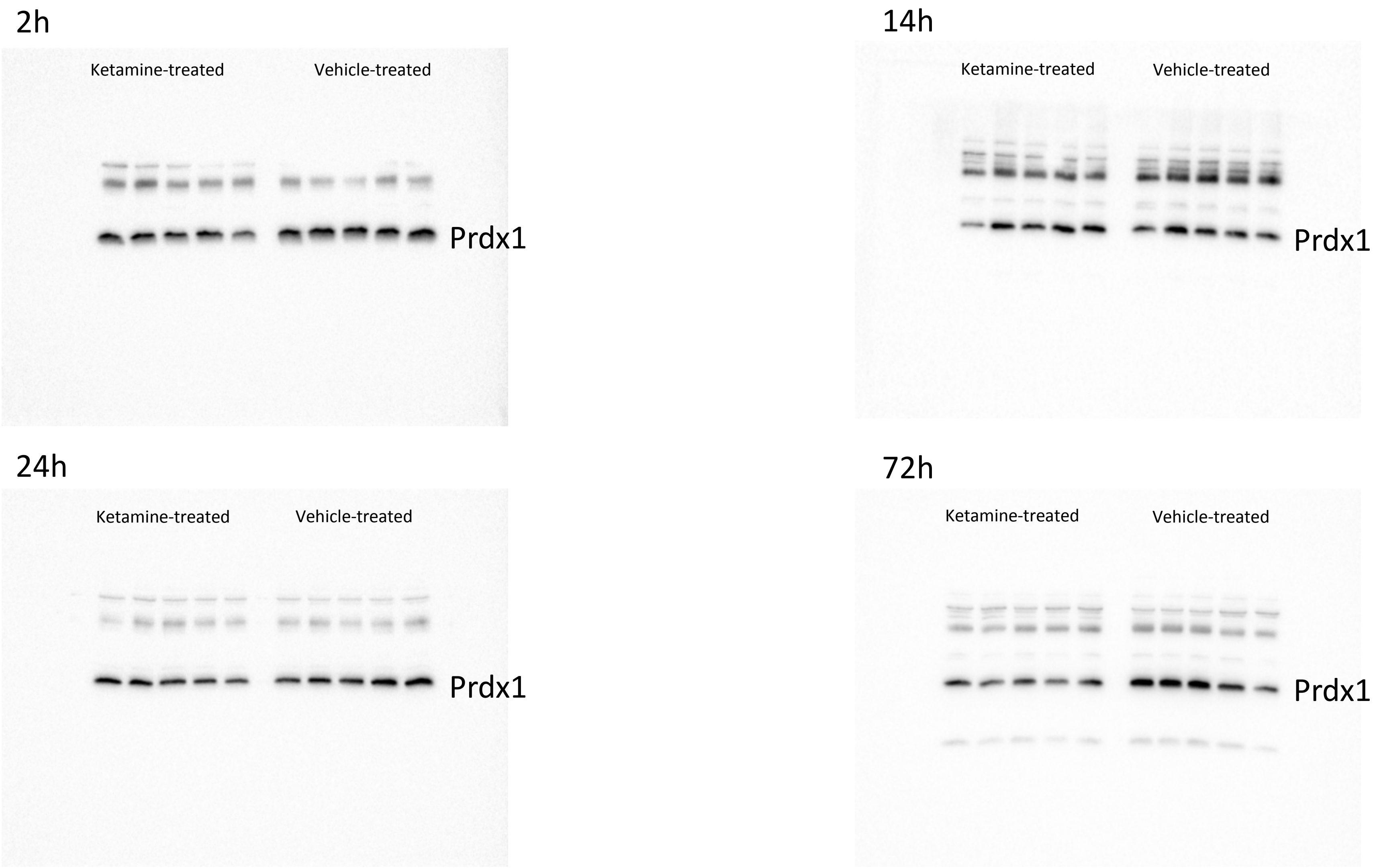

Supplemental figure 1C: Time-dependent Western blot analysis of Peroxiredoxin (Prdx) 1. N=5 mice per group and time point.

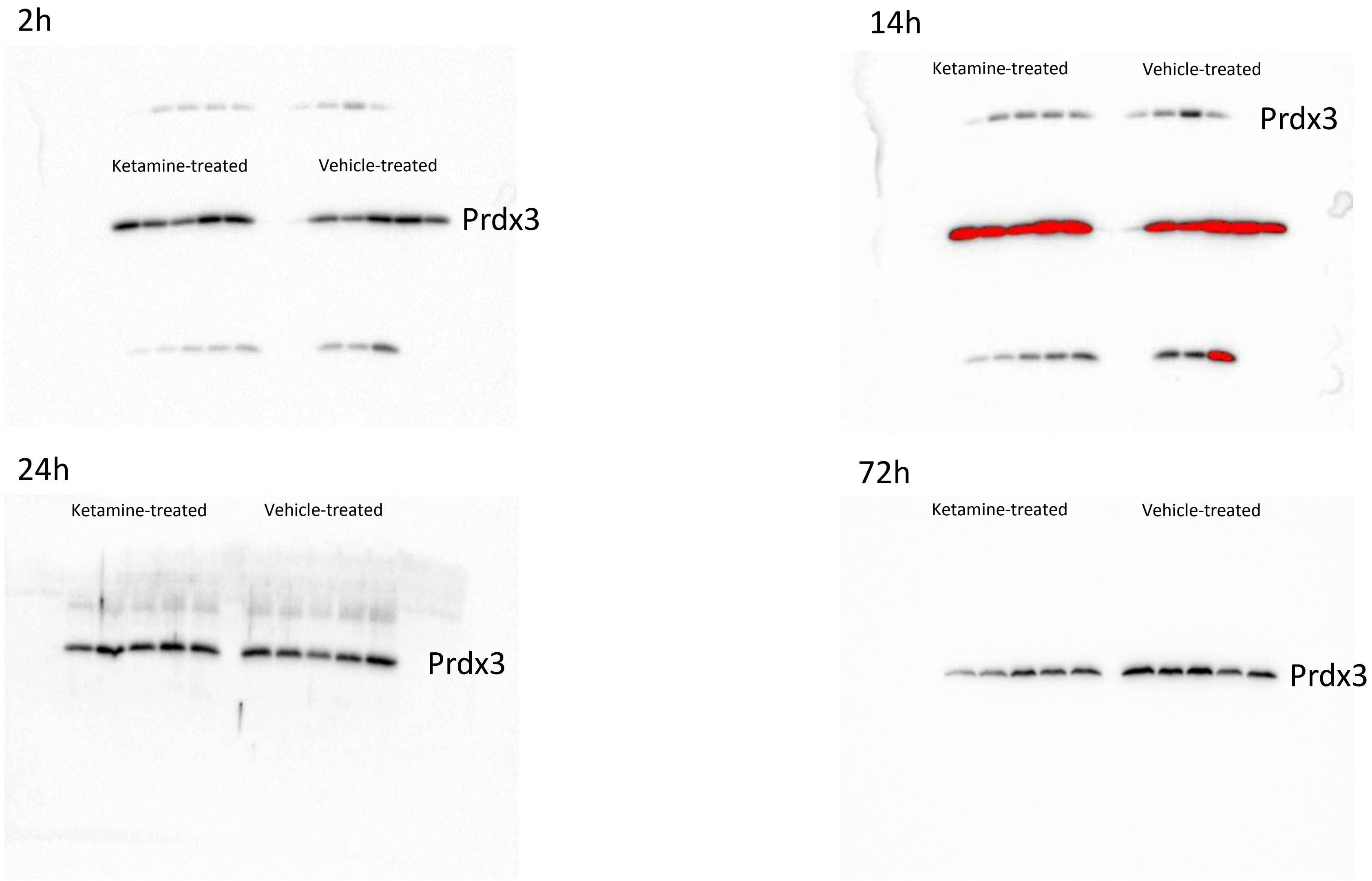

Supplemental figure 1D: Time-dependent Western blot analysis of Peroxiredoxin (Prdx) 3. Blots were cut at the respective band size of Prdx3 and considered for the 2h and 14h time point. N=5 mice per group at the 2h, 24h and 72h time point, n=5 for ketamine-treated and n=4 for vehicle-treated at the 14h time point.
